# Perceived medical disinformation and public trust: Commentary on Grimes and Greenhalgh (2024)

**DOI:** 10.1111/jep.14202

**Published:** 2024-10-18

**Authors:** Brian Baigrie, Mathew Mercuri

**Affiliations:** ^1^ Institute for History and Philosophy of Science and Technology University of Toronto Toronto Ontario Canada; ^2^ Institute of Health Policy, Management and Evaluation University of Toronto Toronto Ontario Canada; ^3^ Department of Medicine McMaster University Hamilton Ontario Canada; ^4^ Institute on Ethics & Policy for Innovation McMaster University Hamilton Ontario Canada; ^5^ Department of Philosophy McMaster University Hamilton Ontario Canada; ^6^ Department of Philosophy University of Johannesburg Johannesburg South Africa

**Keywords:** accountability, COVID‐19, medical disinformation, social media, trust

When people are misled about important topics, whether it results from an honest mistake, negligence, unconscious bias or is intentional (as in the case of disinformation), people can suffer serious harm.^1^ In contrast to an honest mistake, disinformation is disseminated by those who are actively engaged in an attempt to mislead. We have seen the use of disinformation in the context of vaccines, resulting in vaccine refusal, which is a significant threat to public health.^2^, ^3^ A recent article published in this journal by Grimes and Greenhalgh^4^ draws welcome attention to the problem of disinformation in the context of the dissemination of antivaccine disinformation on social media when its purveyors are members of a profession that holds a privileged position of trust with respect to the public, which it is argued amplifies the harm.

Though there is a large body of research examining the influence perceived experts who recommend vaccination have on public uptake, the influence of such experts who act in the opposite role through the dissemination of disinformation has received scant attention (Harris 2024: 2).^2^ Given evidence that vaccine influencers (whether pro or antivaccine) play a disproportionate (and often oversized) role in the public's vaccine uptake, Grimes and Greenhalgh^4^ reasonably contend that the combination of a respected source of information and social media amplifies the direct harm caused by disinformation when people are misled into making decisions respecting the safety of vaccines (i.e., reducing vaccine uptake, elevating individual risk of morbidity and mortality, and increasing the chance of disease outbreaks).^5^, ^6^, ^7^ There is also the indirect harm through the erosion of public trust^8^ and the curbing of critical discussion by physicians and experts who may be concerned about fallout from weighing into online discussion where denialism has been orchestrated and arguably has emboldened people to push back violently against public health.

When we consider this issue in light of a 2023 *JAMA* study,^9^ which showed 52 prominent physicians who disseminated misinformation to large audiences on social media, Grimes and Greenhalgh raise the important question: How should medical regulators respond to the problem of disinformation spread on social media by physicians? The authors mount an argument to the effect that regulatory bodies have an ethical duty to act against doctors who intentionally mislead the public through (e.g.,) the misinterpretation of data, the fabrication of statistics, and sometimes just making things up; i.e., one way to counter the spread of online disinformation is to appeal to governments and relevant regulatory bodies to disincentivize doctors acting as content creators from doing so. There are precedents for such regulation. Legislation is already in place in some jurisdictions, as noted by Edwards^10^ “that regulates content, in whatever medium it appears … laws on data protection, copyright, pornography, spam, race hate, malicious communication, misleading advertising, libel, fraud, equity legislation and image rights.” Misleading advertising violates the trust of the public and undermines the integrity of businesses, and it is not clear that the promotion of unproven Covid‐19 treatments, such as antimalarial drugs, should be treated any differently.

Governments in Canada, the UK, and in Europe have attempted to address the issue of online harm through the introduction of legislation that imposes a duty to act responsibly on social media platforms, including the adoption of policies to identify and mitigate risk, filing a safety plan with a government regulator, and new transparency requirements, which require platforms to publish details on how it addresses harmful content, as well as sharing data with the government and with accredited independent researchers and civil society. Only Europe's Digital Services Act addresses a broad set of social harms, including disinformation and content that undermines democratic elections.

Strategies clearly are needed to deal with the problem of disinformation in the public health sector. A great deal of work remains to understand the scale of antivaccine influencers, who in many ways conduct themselves like biomedical experts; i.e., their online postings typically advance scientific arguments and share scientific links, signalling their expertise in their social media profiles and in their postings. They engage the scientific literature but appear to reject the scientific consensus, even when this consensus (as often was the case during the Covid‐19 pandemic) is a moving target.^2^ Often a substitution of expertise is involved, with content creators trading on their physician or scientist credentials and weighing in on matters where they lack relevant expertise.

Clinicians and scientists should be free to debate issues of clinical care and effectiveness. Indeed, most things we study, including technologies/medicines used in clinical care, leave room for interpretation, or can be better understood with additional investigation. The question is where does reasonable disagreement on interpretation of the data end and what is the appropriate forum for such debate? It is not debate that is problematic—neither clinicians nor scientists should be persecuted for holding a different view when the data allows for such, and the debate is motivated by the spirit of scientific inquiry. What is concerning is the intention to use one's position and status with the public to advance an agenda that is not in the public's best interest. It is that context where advocating a view is not in the spirit of debate, but in the realm of disinformation. The implication is that if we seek to implement a policy to regulate the dissemination of disinformation, it is incumbent that we understand what disinformation is, including the sorts of things that affect the amount of disinformation and how they affect it.^1^ One concern about the authors’ framing of the problem at hand is that while it is the case that disinformation is always misleading, it is not always intended to mislead. Most forms of disinformation, such as lies and propaganda, are misleading because the source intends the information to be misleading. Conspiracy theories and fake alarm calls are misleading because the source systematically benefits (e.g., politically, economically) from their being misleading.^1^ Denialism, as defined by Hoofnagle and Hoofnagle,^11^ and which may be the agenda for the spread of disinformation, is motivated by a desire to undermine a policy, rather than advance understanding of an issue.

For a regulatory policy to work, one needs a mechanism to differentiate the various types of inaccurate information; notably, intentionally misleading information from accidental falsehoods (mistakes). A candidate mechanism fit for the purpose of public health will need to be sensitive to the dynamic nature of science, as evidenced by the evolving understanding of SARS‐CoV‐2's lethality, infectiousness, and constant updating of information, which could be due to epistemic uncertainty (e.g., limits of our data and analysis tools) or evolution of the virus (or both). An effective mechanism will also need to provide a way to assess whether misleading scientific information (whether outdated or incomplete) is to count as misinformation (or as disinformation). The authors pass over this important question quickly but do cite as a consideration “whether statements appear to have been made in good faith” (2024: 4). Many public health service announcements on a range of protective measures, such as social distancing, face coverings, lockdowns, and research studies published during the pandemic could be classified as misinformation but not as disinformation unless one takes the view that there was an intent to mislead the public. One could take the view that public health messaging concerning face coverings, for example, during the pandemic misled the public, but this is not to say that this was the intention behind the mixed messaging on face coverings, nor a justification for the WHO disseminating inaccurate information to the effect that face masks do not confer a benefit over and above social distancing. There may be times that misleading the public can be a prudential strategy towards a further end (such as ensuring that front line health care professionals are prioritised in the allocation of scarce medical resources). Whether this lack of transparency is acceptable to the public is another matter altogether, as there is always a risk it may backfire, leading to erosion of the public's trust in public health, which one could argue happened during the COVID‐19 pandemic.

Given the authors own characterisation of disinformation as misinformation that is spread deliberately (intentionally), how then are regulators to determine whether a vaccine influencer intended to mislead the public through their social media postings? How then are regulators to determine whether the postings were made in good faith? One line of response is that misinformation counts as disinformation if it is not corrected in a timely manner in step with an evolving understanding of the most effective mitigation strategies during a public health emergency. The challenges we highlight should not be seen as a reason to accept disinformation. Rather, we need to think deeply about how to best approach the problem so as to not make matters worse for the public and the advancement of knowledge.

The strategy advanced by Grimes and Greenhalgh^4^ is that one way to counter the spread of online disinformation is to appeal to governments and relevant regulatory bodies to disincentivize purveyors of disinformation from doing so. Online information is largely hosted by global platforms (Google, Facebook), and audience attention is determined by their competing algorithms. The authors pass over a second strategy for regulating disinformation, which involves regulating the platforms that host content that, for example, fuels vaccine hesitancy. The second strategy is to encourage social media companies to modify their platform so that fewer people are exposed to disinformation. These platforms do have the ability to reduce disinformation and to close the accounts of those who are disseminating it. President Trump was “deplatformed” by both Facebook and Twitter both after he declared the US election “stolen” and appeared to incite against democracy. This action was applauded by many, but others were concerned about whether private platforms should have such immense powers to control speech rather than elected governments, or courts. In the past, both the UK and the EU have encouraged the large internet platform companies to self‐regulate by signing them up to a Code of Practice on Disinformation, including closing down fake accounts and demonetising the spread of disinformation.^12^ Another Promising strategy is to impose a “duty of care” in relation to harmful content on social media, which could establish mechanisms to verify academic and professional credentials and the identification of signals within profiles to identify authorities on health‐related topics (Harris 2024, 8).^2^

The authors’ proposal of a policy to disincentivize content creators, rather than police social media platforms, in turn, raises important questions about the scope of regulatory oversight. For example, Lewis^12^ raises the interesting question about the 2‐year delay on the part of the WHO to recognise that SARS‐COV‐2 is airborne, and not transmitted in line with “decades‐old infection‐control teachings about how respiratory viruses generally pass from one person to another”.^12^ The WHO tweeted on March 20, 2021, that “#fact: Covid is NOT airborne,” capitalising the word NOT to highlight their confidence in the truth of this position. It was not until December 2021 that the WHO first used the word airborne on a WHO webpage, stating that “transmission can occur through long‐range airborne transmission.” Lewis points out that the WHO's messaging finally echoed “a chorus of aerosol and public‐health experts had been trying to get it to say since the earliest days of the outbreak”.^12^

The mainstream media frequently focus on the role of the anti‐vaccination movement in reducing vaccine acceptance, and Grimes and Greenhalgh rightly point out that some medical professionals also bear a share of responsibility for fuelling public hesitancy about vaccines and the ongoing erosion of public trust. A commonly held belief is that anti‐vaccination messages lead to reduced acceptance, which leads to reduced coverage which, in turn, causes outbreaks. Even so, it is widely recognised that barriers to high vaccination coverage extend beyond negative messaging about vaccination. The authors note approvingly the WHO's listing of vaccine hesitancy as one of the top ten threats to global health in 2019. Notably, the WHO also included fragile health systems and weak primary care in the list of top threats. These factors also influence vaccine uptake and need to be factored into judgements about the extent of the harm caused by disinformation.

The authors chastise clinicians and scientists who advanced opinions on social media, based on evidence that is “selective, questionable or already refuted.” With the exception of the legalistic suggestion of “a totality of evidence,” there is little by way of suggestion as to what counts as good evidence during a public health emergency when there is an urgent need for novel science on the fly.^13^, ^14^ When the WHO, for example, discounted field epidemiology reports and laboratory‐based aerosol studies (among other sources of evidence for airborne transmission) on the grounds that it was not definitive, did they run afoul of the position that what counts is a totality (preponderance) of evidence? Were they (i.e., the WHO) not selecting data to support their deeply entrenched view about the transmission of respiratory viruses, as Geenhalgh herself has been quoted as conceding?^12^

A final concern is that the policy sketched by Grimes and Greenhalgh is deeply wedded to a top‐down paternalistic approach that overlooks the diverse beliefs, values and trusted sources of information within communities and population subgroups. A third promising strategy that can counter the harm caused by disinformation is to help internet users to modify their online habits in an effort to minimise the chance that they will form false beliefs on the basis of misleading claims. Inoculation theory has been put forward^15^ as a way to reduce susceptibility to misinformation by informing people about how they might be misinformed, but operationalizing this approach has been elusive both at a theoretical level and a practical level.^4^ note that certain demographic groups are more susceptible to disinformation than others, and doubtless fragile health systems and weak primary care are drivers of this susceptibility to disinformation. Helping to mitigate the impact of fragile systems of care on susceptibility to uptake of disinformation may take the form of building partnerships with communities that yield insights into measuring, monitoring, and characterising misinformation that will empower the public to look for signs of deception in the information itself.

Grimes and Greenhalgh raise issues that have important implications on the health of society and our collective ability to implement countermeasures to public health threats. The volume of disinformation and its purported role in spurring hesitancy in or undermining of public health measures, such as vaccines, may imply a lack of systems of accountability. Traditionally, clinicians and scientists have been accountable to their profession or communities. Academic journals and the community of scientists are generally considered the check and balance of misinformation. These activities would take place out of the eyes of the public, and so the impact of “getting it wrong”, whether intentional or by mistake, was often minimal. However, as these discussions have become more public, the traditional mechanisms of accountability are often insufficient. We welcome Grimes and Greenhalgh's call for reconsidering how we ensure accountability. How we do so and who is given the power as the arbitrator of reasonable debate and assessing motivation, however, is not a trivial task. Indeed, admitting we have a problem is the first step.

## CONFLICT OF INTEREST STATEMENT

The authors declare no conflicts of interest.

## ETHICS STATEMENT

This is a commentary, and ethics approval is not required at our institutions.

## Data Availability

This article does not include original data.
